# Effect of moderate alcohol consumption on fetuin-A levels in men and women: post-hoc analyses of three open-label randomized crossover trials

**DOI:** 10.1186/1758-5996-6-24

**Published:** 2014-02-18

**Authors:** Michel M Joosten, Ilse C Schrieks, Henk FJ Hendriks

**Affiliations:** 1 TNO (a Dutch acronym for Netherlands Organisation of Applied Scientific Research), Zeist, the Netherlands; 2 Division of Human Nutrition, Wageningen University, Wageningen, the Netherlands; 3 Department of Internal Medicine, University of Groningen, University Medical Center Groningen, Groningen, the Netherlands

**Keywords:** Alcohol consumption, Fetuin-A, Insulin sensitivity, Liver enzymes, Type 2 diabetes mellitus

## Abstract

**Background:**

Fetuin-A, a liver-derived glycoprotein that impairs insulin-signalling, has emerged as a biomarker for diabetes risk. Although moderate alcohol consumption has been inversely associated with fetuin-A, data from clinical trials are lacking. Thus, we evaluated whether moderate alcohol consumption decreases circulating levels of fetuin-A.

**Methods:**

We analyzed data of three separate open-label, randomized, crossover trials: 1) 36 postmenopausal women consuming 250 ml white wine (25 g alcohol) or white grape juice daily for 6 weeks, 2) 24 premenopausal women consuming 660 ml beer (26 g alcohol) or alcohol-free beer daily for 3 weeks, and 3) 24 young men consuming 100 ml vodka (30 g alcohol) orange juice or only orange juice daily for 4 weeks. After each treatment period fasting blood samples were collected.

**Results:**

Circulating fetuin-A concentrations decreased in men after vodka consumption (Mean ± SEM: 441 ± 11 to 426 ± 11 μg/ml, *p* = 0.02), but not in women after wine (448 ± 17 to 437 ± 17 μg/ml, *p* = 0.16) or beer consumption (498 ± 15 to 492 ± 15 μg/ml, *p =* 0.48) compared to levels after each corresponding alcohol-free treatment. Post-hoc power analyses indicated that the statistical power to detect a similar effect as observed in men was 30% among the postmenopausal women and 31% among the premenopausal women.

**Conclusions:**

In these randomized crossover trials, moderate alcohol consumption decreased fetuin-A in men but not in women. This sex-specific effect may be explained by the relatively short intervention periods or the low statistical power in the trials among women.

**Trials registration:**

ClinicalTrials.gov ID no’s: NCT00285909, NCT00524550, NCT00918918.

## Introduction

Fetuin-A (α-Heremans-Schmid glycoprotein) is an abundant hepatokine that impairs insulin signalling by inhibiting tyrosine kinase activity [1,2]. Several prospective studies have reported positive associations between circulating fetuin-A and type 2 diabetes risk and, concomitantly, observed inverse relations between alcohol consumption and fetuin-A [3-5]. More importantly, a recent case-control study suggested that fetuin-A may partially explain the reduced risk of type 2 diabetes [6] that has consistently been observed with moderate alcohol consumption [7-9]. However, the cross-sectional and observational nature of these alcohol-fetuin-A associations may raise concern about potential confounding. Thus, to comprehensively investigate the effect of moderate alcohol consumption on fetuin-A levels, we performed post-hoc analyses of three randomized crossover interventions with different alcohol-containing beverages in men and women.

## Materials and methods

The rationale of the three trials was to study the effect of moderate alcohol consumption on markers of insulin sensitivity and/or inflammation. Each trial is registered at ClinicalTrials.gov: NCT00285909, NCT00524550, and NCT00918918. Independent medical ethics committees approved the research protocols (The Medical Ethics Committee of the University Medical Centre Utrecht; Utrecht, the Netherlands [NCT00285909] and METOPP; Tilburg, the Netherlands [NCT00524550, and NCT00918918]) and all participants gave written informed consent. Eligible subjects were apparently healthy, were habitual alcohol consumers, refrained from smoking, and had no family history of alcoholism. The design of each individual intervention has been described in more detail elsewhere [10-12]. In short, the three studies were open-label, randomized, crossover intervention trials and were all conducted at TNO (a Dutch acronym for Netherlands Organisation of Applied Scientific Research) in Zeist, the Netherlands. The trials consisted of 1) 36 postmenopausal women consuming 250 ml white wine (25 g alcohol; Chardonnay; Jean d’Alibert, Rieux, France) or white grape juice (Albert Heijn, Zaandam, the Netherlands) daily for 6 weeks between March and June 2006, 2) 24 premenopausal women consuming 660 ml beer (26 g alcohol) or alcohol-free beer daily (both Amstel, Amsterdam, the Netherlands) for 3 weeks between August and November 2007, and 3) 24 young men consuming 100 ml vodka (30 g alcohol; Smirnoff, Diageo, London, UK) and 200 ml orange juice (Appelsientje, Riedel, Ede, The Netherlands) or only orange juice daily for 4 weeks between August and November 2009. Postmenopausal women had an absence of menses for at least two years. Premenopausal women used phase I or II oral contraceptives. Allocation to treatment order (alcohol-containing vs. alcohol-free period) was randomized according to age and body mass index (BMI). After each treatment period, fasting blood samples were obtained. Plasma samples were stored at −80°C (beer and vodka trials) and serum samples at −20°C (wine trial) until analysis. Fetuin-A concentrations were determined by a sandwich enzyme-linked immunosorbent assay (ELISA) (R&D Systems, Minneapolis, MN) with a mean intra-assay coefficient of variation of 6.8%.

Data were analyzed using SAS statistical software (version 8.2; SAS Institute, Cary, NC, USA). Variables were compared between treatments with a mixed analysis of variation (ANOVA) model that included terms for treatment, period and the interaction between period and treatment (indicating possible carryover effects). Correlation coefficients were computed according to Spearman rank order to assess associations between intervention-induced changes in fetuin-A and other biochemical variables. Data are presented as mean ± standard error of the mean (SEM). All tests were two-sided. Statistical significance was defined as *p* < 0.05.

## Results

All subjects completed both arms of their intervention. No notable adverse effects were reported. Age and BMI were 56.5 ± 4.2 y and 25.4 ± 3.3 kg/m^2^ in postmenopausal women, 23.9 ± 4.3 y and 22.2 ± 1.6 kg/m^2^ in the premenopausal women, and 25.5 ± 4.3 y and 22.2 ± 1.6 kg/m^2^ in the men, respectively. Indicators of compliance were the increased high-density lipoprotein (HDL)-cholesterol and adiponectin levels after each of the three alcohol consumption periods compared with after the alcohol-free consumption periods (Table 1).

**Table 1:** Biochemical markers of 24 young men, 24 premenopausal women, and 36 postmenopausal women sampled after an overnight fast after 4, 3 and 6-week treatment periods, respectively, of consuming alcohol-free or alcohol-containing beverages

	**Young men**			**Premenopausal women**			**Postmenopausal women**		
	**Orange juice**	**Vodka and orange juice**	***p*** **value**	**Alcohol-free beer**	**Beer**	***p*** **value**	**White grape juice**	**White wine**	***p*** **value**
Fetuin-A (μg/ml)	441 ± 11	426 ± 11	0.02	498 ± 15	492 ± 15	0.48	448 ± 17	437 ± 17	0.16
Adiponectin (μg/ml)	10.5 ± 1.0	11.8 ± 1.0	0.005	6.8 ± 0.4	7.2 ± 0.4	0.01	12.0 ± 0.7	13.1 ± 0.7	<0.001
Insulin (pmol/l)	59.8 ± 8.5	55.7 ± 8.6	0.53	45.7 ± 4.0	46.1 ± 4.0	0.90	46.5 ± 3.4	40.0 ± 3.4	0.90
Glucose (mmol/l)	5.3 ± 0.10	5.3 ± 0.10	0.76	4.8 ± 0.11	4.8 ± 0.11	0.36	5.4 ± 0.11	5.4 ± 0.11	0.36
HOMA-IR	1.98 ± 0.30	1.87 ± 0.30	0.61	1.41 ± 0.11	1.43 ± 0.11	0.81	1.64 ± 0.13	1.42 ± 0.13	0.02
HDL cholesterol (mmol/l)	1.12 ± 0.05	1.22 ± 0.05	0.009	1.52 ± 0.07	1.62 ± 0.07	0.008	1.57 ± 0.07	1.68 ± 0.07	<0.001
LDL cholesterol (mmol/l)	2.63 ± 0.17	2.70 ± 0.17	0.55	2.40 ± 0.07	2.37 ± 0.07	0.77	3.84 ± 0.12	3.51 ± 0.12	<0.001
Triglycerides (mmol/l)	1.23 ± 0.14	1.33 ± 0.14	0.30	1.27 ± 0.08	1.25 ± 0.08	0.61	1.18 ± 0.08	1.04 ± 0.08	<0.001
Free fatty acids (mmol/l)	0.42 ± 0.03	0.35 ± 0.03	0.07	0.34 ± 0.03	0.29 ± 0.03	0.26	0.43 ± 0.04	0.44 ± 0.04	0.67
Alanine aminotransferase (U/l)	15.2 ± 1.1	15.9 ± 1.1	0.49	10.8 ± 1.8	10.0 ± 1.8	0.21	13.8 ± 2.5	17.4 ± 2.5	0.29
Alkaline phosphates (U/l)	65.1 ± 4.3	65.8 ± 4.3	0.70	56.9 ± 6.5	57.8 ± 6.5	0.68	72.7 ± 2.9	73.6 ± 2.9	0.73
Aspartate aminotransferase (U/l)	21.0 ± 1.0	20.6 ± 1.0	0.62	17.8 ± 2.0	17.8 ± 2.0	0.95	20.9 ± 2.0	24.8 ± 2.0	0.13
γ-Glutamyltransferase (U/l)	19.8 ± 2.4	24.3 ± 2.4	0.003	16.5 ± 2.2	18.5 ± 2.2	0.01	18.4 ± 5.2	27.5 ± 5.2	0.21

Data are presented as means ± SEM. *P* values are obtained from a mixed-model ANOVA. Abbreviations: HOMA-IR homeostasis model assessment of insulin resistance; HDL high-density lipoprotein; LDL low-density lipoprotein.

**Figure 1:**
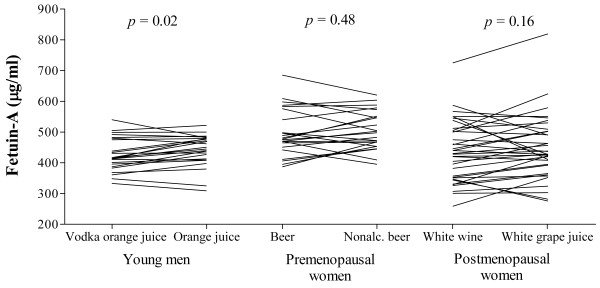
Individual changes of circulating fetuin-A levels at the end of the alcohol or alcohol-free treatment periods after an overnight fast for three open-label randomized crossover trials.

No carry-over effects were found in fetuin-A, indicating that a possible effect on fetuin-A levels due to a treatment given in the first time period of the crossover trial did not persist into the second period and influence the effect of the second treatment. Fetuin-A levels decreased in men after vodka juice consumption (441 ± 11 to 426 ± 11 μg/ml, *p* = 0.02) but not significantly in postmenopausal women after wine (448 ± 17 to 437 ± 17 μg/ml, *p* = 0.16) or in premenopausal women after beer consumption (498 ± 15 to 492 ± 15 μg/ml, *p* = 0.48) (Figure 1) as compared to levels after each corresponding alcohol-free beverage consumption.

No correlations were observed between alcohol-induced changes in fetuin-A and corresponding changes in the homeostasis model assessment of insulin resistance (HOMA-IR) (ρ = 0.01, *p* = 0.95; ρ = 0.25, *p* = 0.26; ρ = 0.20, *p* = 0.24) or changes in adiponectin (ρ = 0.22, *p* = 0.31; ρ = 0.17, *p* = 0.44; ρ = 0.25, *p* = 0.15) among young men, pre- or postmenopausal women, respectively. Changes in HOMA-IR and adiponectin were also not correlated among men (ρ = 0.14, *p* = 0.51), premenopausal women (ρ = 0.01, *p* = 0.96), or postmenopausal women (ρ = 0.27, *p* = 0.11). Also, no consistent correlations were observed between alcohol-induced changes in fetuin-A and analogous changes in fasting blood lipids including HDL-cholesterol and free fatty acids (FFA), or liver function parameters across the three trials.

## Conclusions

In post-hoc analyses of three separate open-label randomized crossover intervention studies, we found that moderate alcohol consumption reduced fetuin-A levels in men but not in women. This decrease was apparent after four weeks of moderate vodka consumption. No consistent correlations between intervention-induced changes in fetuin-A and other biochemical markers were observed across the three studies.

To our knowledge, these are the first intervention studies investigating the effect of different alcohol-containing beverages on circulating fetuin-A. The lowered fetuin-A levels in men after moderate alcohol consumption partially confirm cross-sectional observations in several epidemiological studies [3-6,13,14] and may provide some physiological support for the protective effect of moderate alcohol consumption on the risk of developing type 2 diabetes [6,8] besides adiponectin [15]. Furthermore, these findings extend prior evidence of short-term clinical trials that noted favourable changes in selected biological markers associated with diabetes and cardiovascular risk after moderate alcohol consumption [16].

The underlying physiological explanation how alcohol consumption may lower fetuin-A is not clear. Also, the sex-specific alcohol-fetuin-A effect was unexpected, particularly since all women were either on oral contraceptives or postmenopausal, which limits potential influences of hormonal fluctuations or menstrual cycles. The null finding in our trials among pre- and postmenopausal women do not seem to correspond with a previous observational study among 1331 middle-aged and older US female nurses, where moderate alcohol consumption was inversely associated with plasma fetuin-A even after adjustment for several lifestyle variables, demographic information, and medical history [6]. Perhaps this discrepancy can be explained by the low statistical power in the two trials among women. Post-hoc power analyses indicated that the power to detect a similar effect as observed in men was only 30% among the postmenopausal women and 31% among the premenopausal women.

Circulating fetuin-A was strongly and negatively associated with the insulin-sensitizing adipokine adiponectin in humans [17] and treatment of human adipocytes with fetuin-A repressed *ADIPOQ* mRNA levels [17]. Furthermore, given the prior associations between fetuin-A and insulin resistance [18] and insulin sensitivity [19], we hypothesized that reductions in fetuin-A may play a role in the increased adiponectin levels or improved insulin sensitivity after alcohol consumption [10]. Therefore, we analyzed correlations between intervention-induced changes in fetuin-A and adiponectin levels and other markers of insulin sensitivity, such as HOMA-IR. We, however, did not find such inverse correlations despite the fact that moderate alcohol consumption increased both *ADIPOQ* expression [10] and corresponding circulating adiponectin levels [10-12], suggesting that fetuin-A and adiponectin levels may be independently affected by alcohol. Also, it is important to note that the HOMA-IR index is a weak estimate of insulin resistance, particularly in a small study. The absence of a correlation between alcohol-induced changes in fetuin-A and HOMA-IR may partially be explained by the relatively low FFA levels of the studied participants. In a study among 347 healthy subjects at increased risk of type 2 diabetes, fetuin-A was only inversely associated with insulin sensitivity among individuals with high FFA levels (~ > 0.65 mmol/l) [20].

Strengths of the study are the randomized crossover design (considered the ‘gold standard’ for evidence-based research), the assessment of compliance markers (i.e. HDL-cholesterol and adiponectin) to the study treatments, the inclusion of both sexes, and the broad range of biochemical variables. Some limitations warrant consideration. The trials consisted of alcohol-administration periods of 3 to 6 weeks and were performed among fairly insulin-sensitive subjects. Maybe more profound effects on fetuin-A levels would have been observed if the interventions lasted longer and/or were executed in subjects with glucose levels in the (pre)diabetic range. For example, three months of moderate alcohol consumption decreased fasting glucose levels among subjects with impaired glucose metabolism [21] and fetuin-A levels were particularly associated with an increased diabetes risk among subjects with higher fasting glucose [3,5]. Regardless, the duration of the present interventions were long enough to detect alcohol-induced changes in other biochemical markers such as adiponectin and HDL-cholesterol. Also, the association between moderate alcohol consumption and lower risk of type 2 diabetes mellitus is not limited to subjects with impaired glucose metabolism but also exists for subjects already at low risk for diabetes on the basis of multiple combined low-risk lifestyle behaviours [22]. Nevertheless, the subjects studied were rather lean (mean BMI values 22-26), had no fatty liver (low liver enzyme levels) and were rather insulin sensitive (low HOMA-IR). Also, all premenopausal women used oestrogen-containing oral contraceptives, which may explain their somewhat higher fetuin-A levels given the positive associations between oestrogen and fetuin-A [23,24]. Thus, the data are not representative for a typical at-risk population for metabolic diseases. Second, the daily amounts of alcohol consumed by women (~25 g alcohol) were higher than what is considered ‘moderate’ according to most guidelines (i.e. max. ~15 g alcohol). However, the nadir of the alcohol-diabetes association for women appeared to be at 24 g of alcohol/day in a meta-analysis of 20 prospective studies [8] while alcohol consumption became harmful above 50 g/day (and above 60 g/day for men). Third, post-hoc power analyses showed that there was low statistical power in the two trials among women to detect a similar effect as observed in the trial among men. Fourth, although unlikely since vodka is basically an ethanol-water mixture, we cannot fully exclude a potential beverage-specific effect. Finally, the alcohol-induced reductions in fetuin-A were comparable to associations reported in epidemiological studies [3,5,6], but were relatively small as compared to alcohol’s effect on HDL-cholesterol and adiponectin. It is possible that the findings, including the sex differences, were due to chance.

In conclusion, the results of these three randomized clinical trials with different alcohol-containing beverages demonstrated that short-term moderate alcohol consumption decreases fetuin-A levels in men but not in women. Further research is needed to determine whether long-term moderate alcohol consumption decreases fetuin-A levels. If so, these findings may add to the current knowledge of possible metabolic benefits of moderate alcohol consumption.

## Abbreviations

ANOVA: Analysis of variation; BMI: Body mass index; ELISA: Enzyme-linked immunosorbent assay; FFA: Free fatty acids; HDL-cholesterol: High-density lipoprotein-cholesterol; HOMA-IR: Homeostasis model assessment of insulin resistance; SEM: Standard error of the mean.

## Competing interests

The authors have no potential conflicts relevant to this article.

## Authors’ contributions

MMJ conceived the idea of the study, designed the study, directed the study’s implementation, conducted the statistical analyses, interpreted the data, and wrote the manuscript. ICS assisted in the study’s implementation, assisted in the interpretation of the data, and critically edited the manuscript. HFJH designed the study, directed the study’s implementation, assisted in the interpretation of the data, critically edited the manuscript, and obtained funding. All authors read and approved the final manuscript.

## References

[B1] Goustin AS Abou-Samra AB The "thrifty" gene encoding Ahsg/Fetuin-A meets the insulin receptor: insights into the mechanism of insulin resistance Cell Signal 2011 23 980 990 10.1016/j.cellsig.2010.11.003 21087662

[B2] Stefan N Haring HU The role of hepatokines in metabolism Nat Rev Endocrinol 2013 9 144 152 10.1038/nrendo.2012.258 23337953

[B3] Stefan N Fritsche A Weikert C Boeing H Joost HG Haring HU Schulze MB Plasma fetuin-A levels and the risk of type 2 diabetes Diabetes 2008 57 2762 2767 10.2337/db08-0538 18633113 PMC2551687

[B4] Ix JH Biggs ML Mukamal KJ Kizer JR Zieman SJ Siscovick DS Mozzaffarian D Jensen MK Nelson L Ruderman N Djousse L Association of fetuin-A with incident diabetes mellitus in community-living older adults: the cardiovascular health study Circulation 2012 125 2316 2322 10.1161/CIRCULATIONAHA.111.072751 22511752 PMC3390925

[B5] Laughlin GA Barrett-Connor E Cummins KM Daniels LB Wassel CL Ix JH Sex-specific association of fetuin-A with type 2 diabetes in older community-dwelling adults: the Rancho Bernardo study Diabetes Care 2013 36 1994 2000 10.2337/dc12-1870 23315604 PMC3687317

[B6] Ley SH Sun Q Jimenez MC Rexrode KM Manson JE Jensen MK Rimm EB Hu FB Association between alcohol consumption and plasma fetuin-A and its contribution to incident type 2 diabetes in women Diabetologia 2014 57 93 101 10.1007/s00125-013-3077-8 24105100 PMC3858443

[B7] Koppes LL Dekker JM Hendriks HF Bouter LM Heine RJ Moderate alcohol consumption lowers the risk of type 2 diabetes: a meta-analysis of prospective observational studies Diabetes Care 2005 28 719 725 10.2337/diacare.28.3.719 15735217

[B8] Baliunas DO Taylor BJ Irving H Roerecke M Patra J Mohapatra S Rehm J Alcohol as a risk factor for type 2 diabetes: a systematic review and meta-analysis Diabetes Care 2009 32 2123 2132 10.2337/dc09-0227 19875607 PMC2768203

[B9] Joosten MM Chiuve SE Mukamal KJ Hu FB Hendriks HF Rimm EB Changes in alcohol consumption and subsequent risk of type 2 diabetes in men Diabetes 2011 60 74 79 10.2337/db10-1052 20876712 PMC3012199

[B10] Joosten MM Beulens JW Kersten S Hendriks HF Moderate alcohol consumption increases insulin sensitivity and ADIPOQ expression in postmenopausal women: a randomised, crossover trial Diabetologia 2008 51 1375 1381 10.1007/s00125-008-1031-y 18504547 PMC2491412

[B11] Joosten MM Witkamp RF Hendriks HF Alterations in total and high-molecular-weight adiponectin after 3 weeks of moderate alcohol consumption in premenopausal women Metabolism 2011 60 1058 1063 10.1016/j.metabol.2011.01.001 21353262

[B12] Joosten MM van Erk MJ Pellis L Witkamp RF Hendriks HF Moderate alcohol consumption alters both leucocyte gene expression profiles and circulating proteins related to immune response and lipid metabolism in men Br J Nutr 2012 108 620 627 10.1017/S0007114511005988 22142458

[B13] Weikert C Stefan N Schulze MB Pischon T Berger K Joost HG Haring HU Boeing H Fritsche A Plasma fetuin-A levels and the risk of myocardial infarction and ischemic stroke Circulation 2008 118 2555 2562 10.1161/CIRCULATIONAHA.108.814418 19029462

[B14] Jensen MK Bartz TM Mukamal KJ Djousse L Kizer JR Tracy RP Zieman SJ Rimm EB Siscovick DS Shlipak M Ix JH Fetuin-A, type 2 diabetes, and risk of cardiovascular disease in older adults: the cardiovascular health study Diabetes Care 2013 36 1222 1228 10.2337/dc12-1591 23250801 PMC3631840

[B15] Beulens JW Rimm EB Hu FB Hendriks HF Mukamal KJ Alcohol consumption, mediating biomarkers, and risk of type 2 diabetes among middle-aged women Diabetes Care 2008 31 2050 2055 10.2337/dc08-0814 18628567 PMC2551653

[B16] Brien SE Ronksley PE Turner BJ Mukamal KJ Ghali WA Effect of alcohol consumption on biological markers associated with risk of coronary heart disease: systematic review and meta-analysis of interventional studies BMJ 2011 342 d636 10.1136/bmj.d636 21343206 PMC3043110

[B17] Hennige AM Staiger H Wicke C Machicao F Fritsche A Haring HU Stefan N Fetuin-A induces cytokine expression and suppresses adiponectin production PLoS One 2008 3 e1765 10.1371/journal.pone.0001765 18335040 PMC2258416

[B18] Stefan N Hennige AM Staiger H Machann J Schick F Krober SM Machicao F Fritsche A Haring HU Alpha2-Heremans-Schmid glycoprotein/fetuin-A is associated with insulin resistance and fat accumulation in the liver in humans Diabetes Care 2006 29 853 857 10.2337/diacare.29.04.06.dc05-1938 16567827

[B19] Mori K Emoto M Yokoyama H Araki T Teramura M Koyama H Shoji T Inaba M Nishizawa Y Association of serum fetuin-A with insulin resistance in type 2 diabetic and nondiabetic subjects Diabetes Care 2006 29 468 10.2337/diacare.29.02.06.dc05-1484 16443916

[B20] Stefan N Haring HU Circulating fetuin-A and free fatty acids interact to predict insulin resistance in humans Nat Med 2013 19 394 395 10.1038/nm.3116 23558619

[B21] Shai I Wainstein J Harman-Boehm I Raz I Fraser D Rudich A Stampfer MJ Glycemic effects of moderate alcohol intake among patients with type 2 diabetes: a multicenter, randomized, clinical intervention trial Diabetes Care 2007 30 3011 3016 10.2337/dc07-1103 17848609

[B22] Joosten MM Grobbee DE van der AD Verschuren WM Hendriks HF Beulens JW Combined effect of alcohol consumption and lifestyle behaviors on risk of type 2 diabetes Am J Clin Nutr 2010 91 1777 1783 10.3945/ajcn.2010.29170 20410096

[B23] Laughlin GA Cummins KM Wassel CL Daniels LB Ix JH The association of fetuin-A with cardiovascular disease mortality in older community-dwelling adults: the Rancho Bernardo study J Am Coll Cardiol 2012 59 1688 1696 10.1016/j.jacc.2012.01.038 22554599 PMC3345127

[B24] Rasul S Ilhan A Reiter MH Todoric J Farhan S Esterbauer H Kautzky-Willer A Levels of fetuin-A relate to the levels of bone turnover biomarkers in male and female patients with type 2 diabetes Clin Endocrinol (Oxf) 2012 76 499 505 10.1111/j.1365-2265.2011.04246.x 21958193

